# Dynamic FoxP2 levels in male zebra finches are linked to morphology of adult-born Area X medium spiny neurons

**DOI:** 10.1038/s41598-020-61740-6

**Published:** 2020-03-16

**Authors:** Jennifer Kosubek-Langer, Constance Scharff

**Affiliations:** Department of Animal Behavior, Institute of Biology, Freie Universität Berlin, Berlin, Germany

**Keywords:** Neural circuits, Adult neurogenesis, Social behaviour, Spine plasticity

## Abstract

The transcription factor FOXP2 is crucial for the formation and function of cortico-striatal circuits. *FOXP2* mutations are associated with specific speech and language impairments. In songbirds, experimentally altered FoxP2 expression levels in the striatal song nucleus Area X impair vocal learning and song production. Overall FoxP2 protein levels in Area X are low in adult zebra finches and decrease further with singing. However, some Area X medium spiny neurons (MSNs) express FoxP2 at high levels (FoxP2^high^ MSNs) and singing does not change this. Because Area X receives many new neurons throughout adulthood, we hypothesized that the FoxP2^high^ MSNs are newly recruited neurons, not yet integrated into the local Area X circuitry and thus not active during singing. Contrary to our expectation, FoxP2 protein levels did not predict whether new MSNs were active during singing, assayed via immediate early gene expression. However, new FoxP2^high^ MSNs had more complex dendrites, higher spine density and more mushroom spines than new FoxP2^low^ MSNs. In addition, FoxP2 expression levels correlated positively with nucleus size of new MSNs. Together, our data suggest that dynamic FoxP2 levels in new MSNs shape their morphology during maturation and their incorporation into a neural circuit that enables the maintenance and social modulation of adult birdsong.

## Introduction

The forkhead box P2 transcription factor (FOXP2) is linked to speech and language disorders. Heterozygous *FOXP2* mutations in humans affect both the coordination of fine orofacial movements and language perception^1–3^. Because songbirds – like humans – need to learn most of their communicative vocalizations, they offer a unique model to study the role of FoxP2 (for nomenclature FOXP2/FoxP2 see Methods) for vocal learning and for the maintenance of learned vocalizations as adults^4^. Studying the relationship between FoxP2 and vocal learning in songbirds may inform the neurogenetic mechanism underlying the speech deficits in patients carrying FOXP2 mutations for the following reasons. The *FoxP2* protein coding sequence is highly conserved between humans and songbirds as are the brain expression patterns, notably in the cerebellum and striatum^5–7^. Moreover, genetic manipulations of FoxP2 expression levels in the striatal song nucleus Area X during the critical phase of song learning lead to inaccurate and incomplete imitation of the tutor’s song and more variable vocal production^3,8–10^. This phenotype bears similarities to the specific speech deficits called developmental verbal dyspraxia, DVD (or childhood apraxia of speech), that patients carrying *FOXP2* mutations suffer from. The core-phenotype of DVD consists of altered precision, consistency and sequencing of movements underlying speech in the absence of neuromuscular deficits^11^. In addition, altered FoxP2 levels in adult Area X affect the dopaminergic modulation of corticostriatal signaling important to song variability and affect song maintenance^12,13^, stressing the fact that tight regulation of FoxP2 expression is a prerequisite for correct neural transmission in differentiated neural circuits. Additional effects of Foxp2 and its disruption on the embryonic development and the function of neural circuits have been described in mice^14–23^. Further evidence for the biological relevance of tight regulation of FoxP2 expression levels comes from the following studies. FoxP2 expression levels in Area X transiently increase during song learning but are lower in adults^7,24^. Singing decreases overall FoxP2 levels in Area X but not in the surrounding striatum and the degree of FoxP2 down regulation correlates with the amount of produced song^25–27^. This relationship is missing in deafened birds, pointing to an important role of auditory feedback for singing-driven FoxP2 down regulation^27^. How does singing affect FoxP2 expression at the cellular level? Medium spiny neurons (MSNs), the most abundant cell type in the avian striatum, predominantly express FoxP2 at low levels (FoxP2^low^) while a subset expresses FoxP2 at very high levels (FoxP2^high^). Both subtypes are not equally affected by singing; the density of FoxP2^high^ MSNs is not measurably different after singing, contrary to the decreasing density of FoxP2^low^ MSNs^24^. The authors hypothesized that the difference might be due to the neuronal age. Adult Area X constantly receives new MSNs that originate at the ventricular zone^28–33^. FoxP2^high^ MSNs colocalize more frequently with a marker for new neurons than FoxP2^low^ MSNs^24^. Recently we showed that new MSNs mature and participate in singing activity – as measured by immediate early gene activation – within a timeframe of six weeks^34^. Whether FoxP2 influences not only the function but also the integration of new neurons into existing circuits is still an open question. Based on the results of Thompson *et al*. (ref. ^21^) we hypothesized that FoxP2^high^ MSNs are newly recruited into Area X and need to become FoxP2^low^ MSNs before they can participate in singing. To test this, we labelled neuronal progenitors in adult zebra finches. At different time points after these cells had migrated into Area X, we quantified their expression levels of FoxP2 and whether they also expressed the immediate early gene expression EGR-1 after singing.

In rodents, Foxp2 expression is associated with neurite outgrowth, neuronal morphology and synapse formation in cortico-striatal circuits^18,19,35–37^. Foxp2 expression levels vary in striatal MNSs and these differences may be relevant for the morphology of striatal MSNs. Dopamine receptor 1 (D1) expressing MSNs^38^ express Foxp2 at higher levels than dopamine receptor 2 (D2) expressing MSNs^23,35^. These differences in Foxp2 levels may be linked to anatomical differences between D1 and D2 MSNs^35,39^. Furthermore, in mice carrying humanized Foxp2 alleles (Foxp2^hum/hum^ mice), Foxp2^high^ MSNs are more numerous in the dorsal striatum and their MSNs have longer dendrites than wildtype mice^36,37^. Based on the latter results we hypothesized that FoxP2 levels of new MSNs in adult songbirds correlate with their neural morphology. To test this, we virally labelled neural progenitors and analyzed their FoxP2 expression, dendrite complexity and spine density after migration into Area X.

## Results

### Dynamic FoxP2 levels in new MSNs in Area X

**Figure 1:**
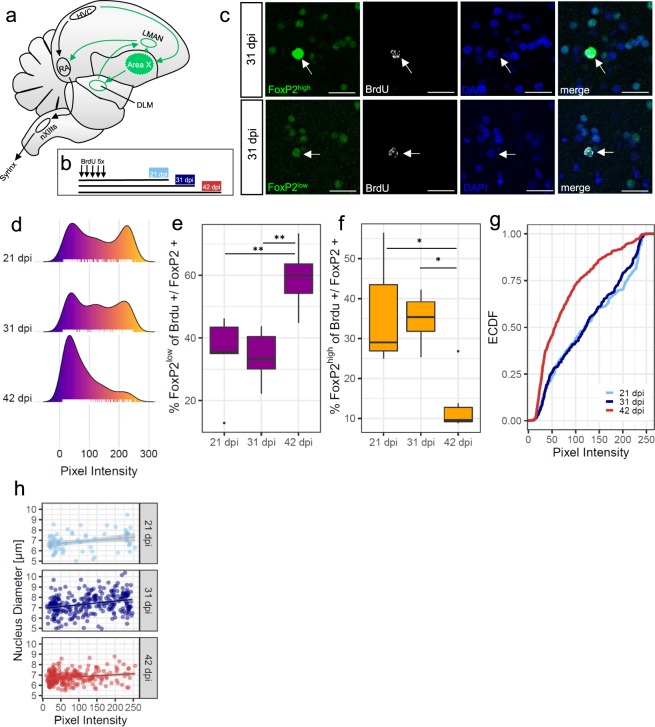
Dynamic FoxP2 expression levels in new MSNs. (**a**) The song motor pathway (main nuclei outlined in black) controls the vocal organ (syrinx). The anterior forebrain pathway (shown in green) forms a cortico (HVCm, lMAN) -basal ganglia (Area X) –thalamo (DLM) –cortical (RA) loop. (**b**) Experimental schedule: adult male zebra finches received BrdU on five consecutive days and were sacrificed 21, 31 or 42 days after injections (dpi). (**c**) Examples of FoxP2^high^ (top row, immunoreactivity shown in green) and FoxP2^low^ (bottom row) new (BrdU+ immunoreactivity shown in white) MSNs in Area X at 31 dpi. Nuclear expression of FoxP2 and BrdU coincides with DAPI label in blue. (**d**) Density plots of FoxP2 pixel intensities of individual new MSNs in Area X at different time points after BrdU injections. The color scheme indicates increasing pixel intensity from low (blue) to high (yellow) intensity. Ticks at the bottom of each plot represent individual MSNs. (**e**) Percentage of new FoxP2^low^ MSNs significantly increases from 21/31 dpi to 42 dpi. (**f**) Percentage of new FoxP2^high^ MSNs significantly decreases from 21/31 dpi to 42 dpi. (**g**) Empirical cumulative distribution function (ECDF) of FoxP2 pixel intensities of individual new MSNs in Area X. FoxP2 pixel intensities are similar at 21 and 31 dpi and are lower at 42 dpi. (**h**) FoxP2 pixel intensities of new MSNs positively correlate with nucleus diameter. Each dot represents one new MSNs. Sample size (d-h): 733 MSNs of 17 zebra finches. *p ≤ 0.05, **p ≤ 0.01. Scale bar: 20 µm (**g**). RA, Robust nucleus of the arcopallium; LMAN, Lateral magnocellular nucleus of the anterior nidopallium; NXIIts, tracheosyringeal part of the hypoglossal nucleus; DLM, Dorsal lateral nucleus of the medial thalamus.

To assess FoxP2 protein levels in individual newborn neurons we labelled progenitor cells with Bromodeoxyuridine (BrdU) and detected BrdU+/FoxP2+ cells after 21, 31 and 42 days post BrdU injection (dpi) in Area X of adult male zebra finches (Fig. 1a,b). We found that FoxP2 expression in Area X was very variable, with some neurons expressing FoxP2 at particularly high levels and some at low levels (Fig. 1c). At 21 dpi and at 31 dpi, the mean pixel intensities of all BrdU+/FoxP2+ cells formed a bimodal distribution, whereas at 42 dpi the distribution was unimodal and shifted to low FoxP2 expression (Fig. 1d). We classified all neurons that had expression intensities within the top 30% of the measured pixel intensity distribution as FoxP2^high^ neurons. Neurons within the bottom 30% of the measured pixel intensity distribution were considered as FoxP2^low^. Because we were interested in the two extremes of the expression levels in this study, we did not analyze the new neurons with intermediate FoxP2 expression levels further (29.3% ± 9.4, SD, see Methods). At 21 dpi and at 31 dpi 36.17% ± 6.02 (SEM) and 34.91% ± 2.53 (SEM) percent of all BrdU+ cells were FoxP2^high^ neurons, respectively. At 42 dpi the percentage of FoxP2^high^ cells had significantly decreased to 12.95% ± 2.87 (SEM) on average (p = 0.0077, Kruskal-Wallis test, Fig. 1f,g). The percentage of FoxP2^low^ neurons increased significantly from 21 dpi (34.59 ± 5.86, SEM) and 31 dpi (34.06 ± 3.35, SEM) to 42 dpi (59.19 ± 4.02, SEM, p = 0.013, ANOVA, Fig. 1e,g). We also noticed that BrdU+ cells varied in their nucleus size. Quantification revealed that the distribution of the nucleus size was shifted towards bigger nuclei at 31 dpi (data not shown). BrdU+/FoxP2+ cells at 31 dpi had significantly bigger nuclei (7.37 µm ± 0.94 (SD) than BrdU+/FoxP2+ cell at 21 dpi (6.88 µm ± 0.9 (SD), p = 0.00025, chi-square = 75.358, df = 2) or 42 dpi (6.74 µm ± 0.59 (SD), p = 2 × 10^−6^, chi-square = 75.358, df = 2, data not shown). Interestingly there was a significant positive relationship with a low effect size between nucleus size and FoxP2 expression levels in all three experimental groups (21 dpi: r^2^ = 0.086, p = 0.017, 31 dpi: r^2^ = 0.083, p = 5.2 × 10-7, 42 dpi = r^2^ = 0.039, p = 0.0012, Fig. 1h).

### Singing induced activity of new MSNs is independent of FoxP2 levels

In adult zebra finches FoxP2 expression levels in Area X are behaviourally regulated. Undirected singing leads to downregulation of FoxP2 mRNA and protein^24–26^. Undirected singing is also associated with expression of the immediate early gene *EGR-1* in Area X, which is therefore often used as a molecular readout of the neuronal activity associated with undirected song^40–44^. We hypothesized that FoxP2 levels in new FoxP2^high^ neurons needed to be downregulated before activation by singing could occur, resulting in EGR-1 expression. Consequently, we did not expect to find BrdU+/FoxP2^high^/EGR-1+ MSNs in Area X. To test this, we analyzed BrdU+/EGR-1 ± /FoxP2+ cells in birds that had sung before sacrifice after 21 dpi, 31 dpi and 42 dpi.

**Figure 2:**
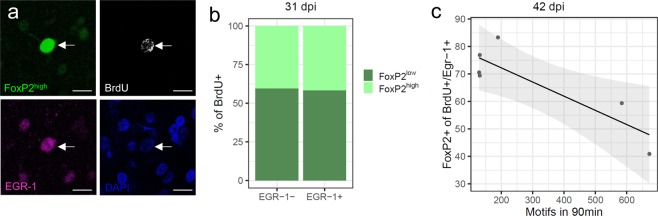
Singing-induced EGR-1 activation of new MSNs is independent of FoxP2 levels. (**a**) The white arrow in all 4 panels points to a new (BrdU+ immunoreactivity, white) MSN that expresses FoxP2^high^ (immunoreactivity, green) and also EGR-1 (immunoreactivity, purple) after undirected singing. The blue DAPI staining shows other cells that are not new, but express FoxP2 at low levels, some of which also express EGR-1. Scale bar: 10 µm. (**b**) At 31 dpi new neurons can either be activated by undirected singing (EGR-1+, right column) or not (EGR-1-, left column). In both cases, the new MSN can either express FoxP2^low^ or FoxP2^high^. (**c**) At 42 dpi the number of FoxP2/BrdU+/EGR-1+ neurons negatively correlate with the number of motifs sung during the 90 min before sacrifice. Sample size (**b**): 108 MSNs of 6 zebra finches. Sample size (**c**): 156 MSNs of 6 zebra finches.

Contrary to our hypothesis we found BrdU+/FoxP2^high^/EGR-1+ cells in Area X (Fig. 2a) in all groups, with large differences between birds. At 31 dpi, on average 41.45% ± 15.43 (SEM) of new neurons expressed FoxP2^high^ and were also activated by singing (BrdU+/FoxP2^high^/EGR-1+) and 40.28% ± 13.34 (SEM) of the new neurons that expressed FoxP2^high^ were not activated by singing (BrdU+/FoxP2^high^/EGR-1-) (Fig. 2b). At 42 dpi, the more birds had sung the fewer FoxP2^high^ new neurons were found, resulting in a significant negative relationship between the number of BrdU+/FoxP2^+^/EGR-1+ neurons and the number of motifs sung before sacrifice (r^2^ = 0.753, p = 0.025, Fig. 2c) which was not the case at 21 dpi (r^2^ = 0.168, p = 0.493, data not shown) nor at 31 dpi (r^2^ = 0.164, p = 0.425, data not shown).

### FoxP2 levels in new MSNs influence dendritic arborization and spine density

**Figure 3:**
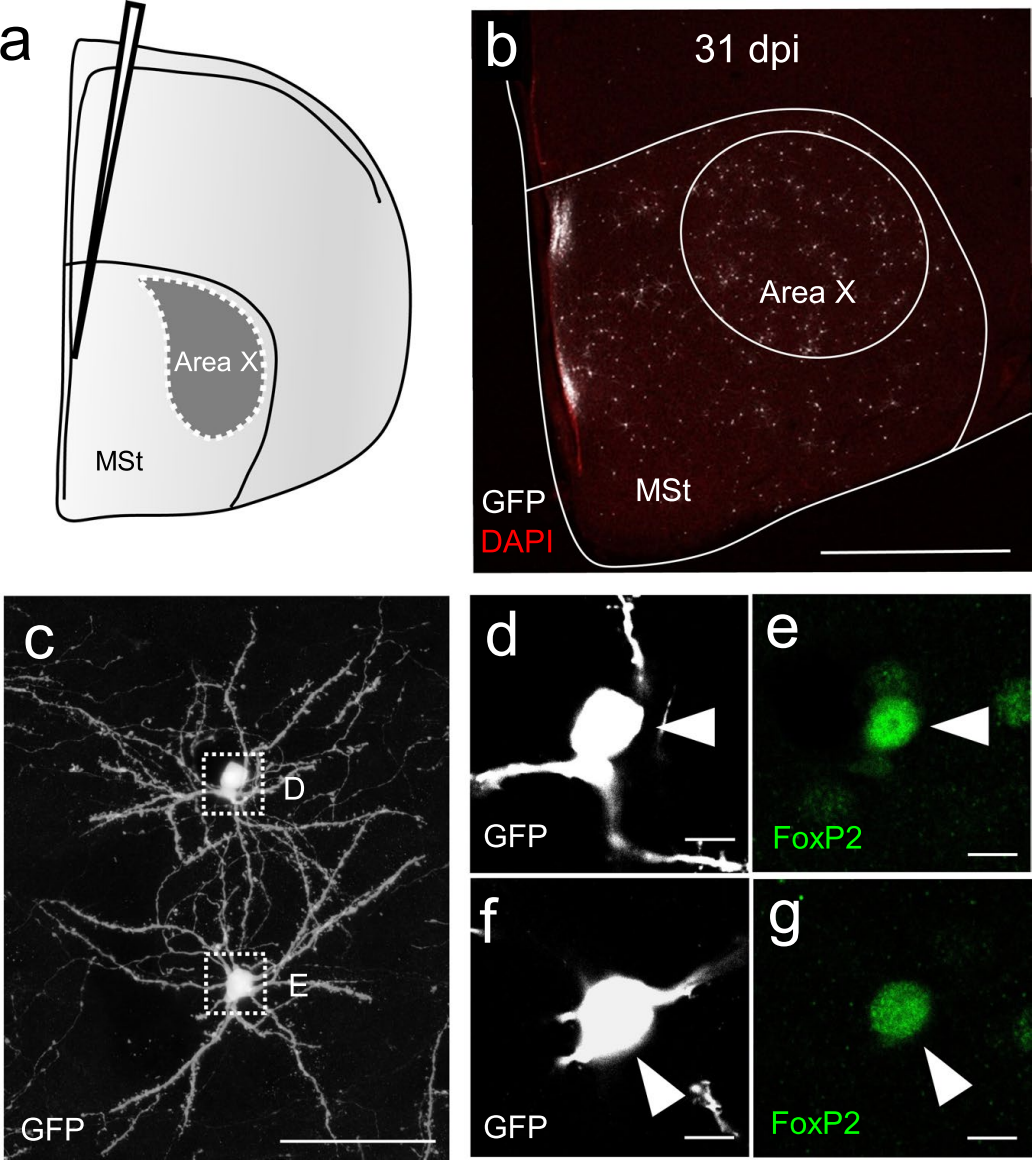
New MSNs that were GFP-labeled as progenitors at the ventricular zone and migrated to Area X express FoxP2. (**a**) Injections with a lentivirus into the ventricular zone resulted in GFP expression in the neural progenitors of MSN. (**b**) 31 dpi after viral injections, many GFP-labelled neurons can be observed in Area X and the surrounding striatum. (**c**) New GFP-expressing MSNs in Area X. Somata in the dashed boxes are magnified in (**d**) and (**e**). (**d**,**e**) Virally labelled MSNs (GFP immunoreactivity, white) express FoxP2 (immunoreactivity, green). Scale bars: 10 µm (d, e), 50 µm (**c**), 1 mm (**b**).

**Figure 4:**
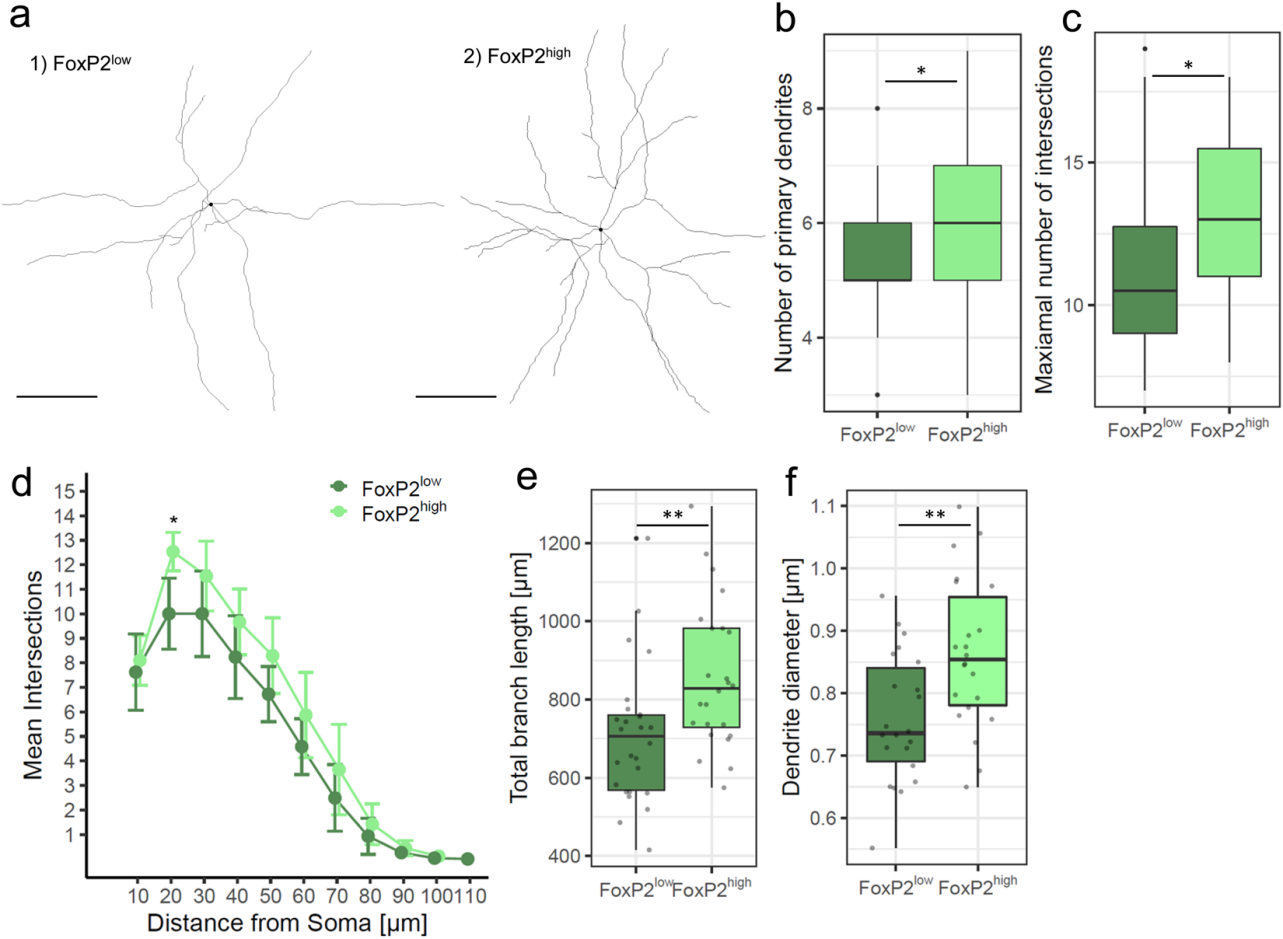
FoxP2 expression levels are linked to distinct morphologies of new MSNs at 31 dpi. (**a**) Examples of dendrite tracings of new FoxP2^low^ and FoxP2^high^ MSNs. The black dot marks the center of the soma. (**b**) Number of primary dendrites is significantly increased in FoxP2^high^ compared to FoxP2^low^ new MSN. (**c**) Maximal number of intersections between dendrites and Sholl circles is significantly higher in FoxP2^high^ than in FoxP2^low^ new MSN. (**d**) Sholl analysis revealed that dendrites of new FoxP2^high^ MSNs had more complex arborizations as indicated by more intersections at 20 µm from the soma than new FoxP2^low^ MSNs. Shown are mean ± SEM. Data points of FoxP2^high^ neurons were slightly shifted to the right for better visibility. (**e**) FoxP2^high^ new MSNs have a significantly higher total branch length than FoxP2^low^ new MSNs. (**f**) At 31 dpi new FoxP2^high^ MSNs have thicker dendrites than new FoxP2^low^ MSNs. Sample size (a-f): 52 MSNs of 4 zebra finches. Scale bars: 25 µm. *p ≤ 0.05; **p ≤ 0.01.

**Figure 5:**
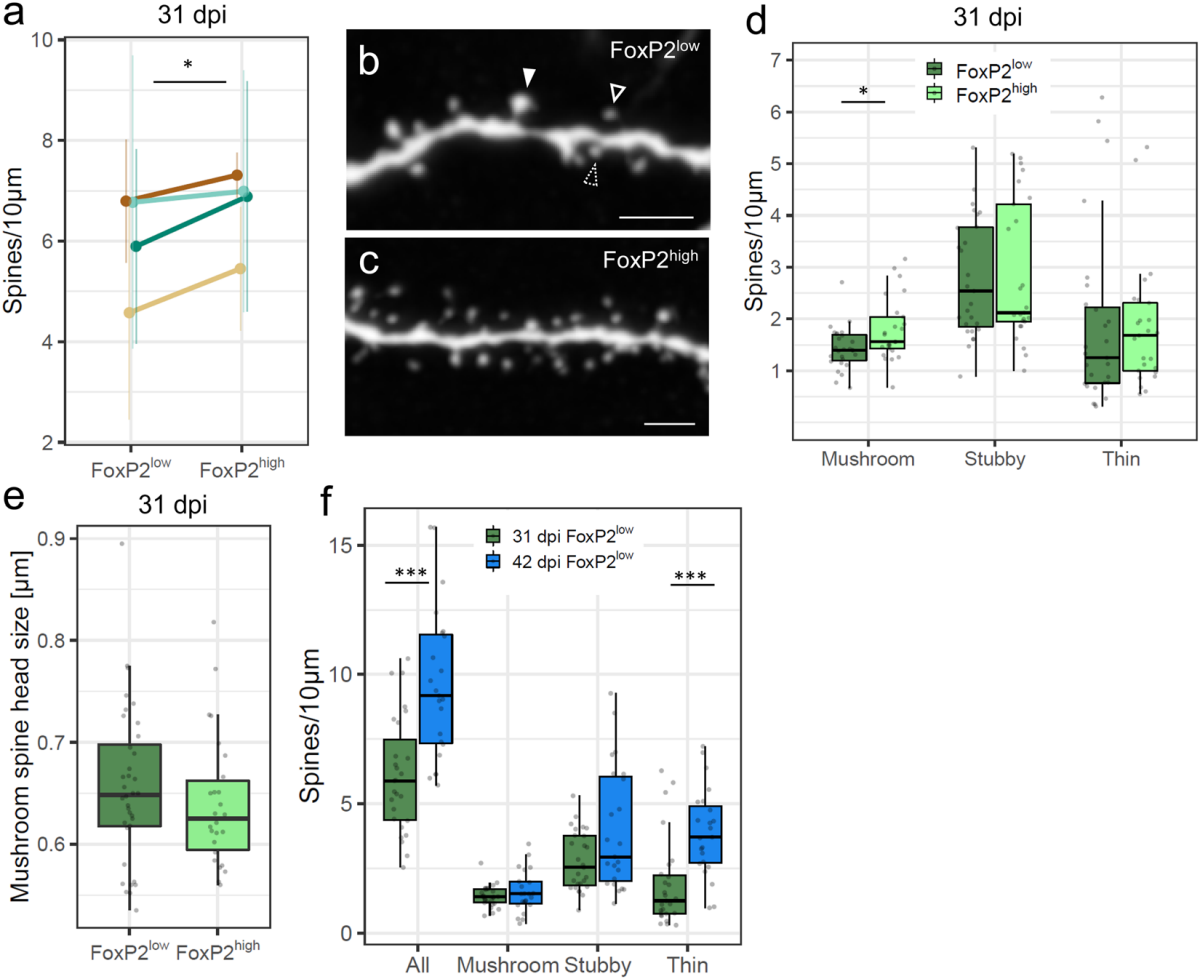
FoxP2 levels are associated with dendritic spine density of new MSNs. (**a**) New FoxP2^high^ MSNs had significantly more dendritic spines than new FoxP2^low^ MSNs at 31 dpi (shown are mean ± SEM). Lines connect data from the same animal. (**b**) Confocal 12 µm projection showing an example of FoxP2^low^ new MSN dendrite with mushroom (filled arrow), stubby (dashed arrow) and thin spines (unfilled arrow). (**c**) Example of FoxP2^high^ new MSNs dendrite. (**d**) New FoxP2^high^ MSNs have more mushroom spines than FoxP2^low^ new MSN at 31 dpi. (**e**) Mushroom spine head size is not different between MSNs with different FoxP2 levels. (**f**) New FoxP2^low^ MSNs at 42 dpi show overall more dendritic spines and more thin spines than new FoxP2^low^ MSNs at 31 dpi. Sample size (**a**,**d**,**e**,**f**): 52 MSNs of 4 zebra finches. Sample size (**f**): 23 MSNs of 3 zebra finches. *p ≤ 0.05; ***p ≤ 0.001. Scale bars: 5 µm (**b**,**c**).

Because of the relationship of FoxP2 levels and nucleus size we wanted to further characterize the morphology of new neurons expressing different FoxP2 levels. We used a lentiviral approach to express Green Fluorescent Protein (GFP) in progenitor cells at their place of birth in the lateral wall of the lateral ventricle (Fig. 3a). After 31 and 42 dpi we found labeled neurons (GFP+) in Area X and the surrounding striatum (Fig. 3b). GFP+ neurons exhibited the typical morphology of medium spiny neurons with small somata and spiny dendrites (Fig. 3c) and expressed FoxP2 (Fig. 3d,e). First, we traced FoxP2^high^ and FoxP2^low^ new neurons at 31 dpi (Fig. 4a) and found that FoxP2^high^ neurons had more primary dendrites (p = 0.021, Mann Whitney test, Fig. 4b), a higher total branch length (p = 0.003, Mann Whitney test, Fig. 4e) and thicker dendrites than FoxP2^low^ neurons (p = 0.003, t-test, Fig. 4f). Second, we analyzed the extent of dendritic arborization of GFP+/FoxP2+ neurons using a Sholl analysis (see Methods). At 31 dpi FoxP2^high^ neurons had more intersections at 20 µm distance from the soma (p = 0.024, paired t-test, Fig. 4a,d) and a higher number of maximal intersections (p = 0.019, t-test, Fig. 4c) than FoxP2^low^ neurons, reflecting more extensive dendritic arborizations in FoxP2^high^ than FoxP2^low^ neurons. Second, Third, we used a semi-automated quantification approach to assess the number of dendritic spines in GFP+/FoxP2+ neurons at 31 dpi. FoxP2^high^ neurons in Area X had more dendritic spines than FoxP2^low^ neurons (p = 0.034, paired t-test, Fig. 5a–c). Overall, FoxP2^high^ neurons had more mushroom spines than FoxP2^low^ neurons (p = 0.0186, Mann Whitney test, Fig. 5d). There was no difference in the number of stubby spines (p = 0.819, Mann Whitney test) or thin spines (p = 0.409, Mann Whitney test) between FoxP2^high^ and FoxP2^low^ neurons (Fig. 5d). Because of the difference in mushroom spine number, we assumed that FoxP2 expression levels might influence mushroom spine head size, too. However, quantification revealed no difference in mushroom spine head size between FoxP2^high^ and FoxP2^low^ neurons (p = 0.317, Mann Whitney test, Fig. 5e). In a last step we compared spine densities between new MSNs at 31 dpi and 42 dpi. Since at 42 dpi only few new neurons were FoxP2^high^ we included only FoxP2^low^ new neurons in this analysis (Fig. 5f). At 42 dpi, the spine density of FoxP2^low^ new neurons was higher than in FoxP2^low^ neurons at 31 dpi (p = 2.517 × 10^−4^, t-test, Fig. 5f). This elevated spine density was largely due to an increase in thin spines (p = 8.422 × 10^−4^, Mann Whitney test) and not mushroom spines (p = 0.39, t-test) or stubby spines (p = 0.119, Mann Whitney test).

## Discussion

**Figure 6:**
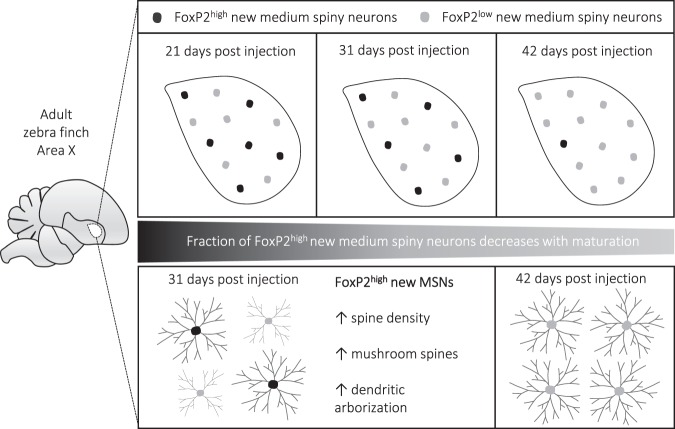
Graphical summary of the main results. The fraction of adult generated FoxP2^high^ MSNs from zebra finch Area X decreases with maturation. FoxP2^high^ new MSNs show higher spine densities, more mushroom spines and a more complex dendritic arborization than FoxP2^low^ new MSNs.

In the present study we investigated the dynamics of FoxP2 expression in adult-born MSNs in the striatal song nucleus Area X of adult male zebra finches. We show that the new MSN strongly expressed FoxP2 at their arrival in Area X from the ventricular zone (VZ) where they were born 21 days prior. During this stage and at intermediate maturation stages (31 days) one third of new MSNs expressed FoxP2 at high levels. At the late maturation stage (42 days) most new MSNs expressed FoxP2 at low levels (Fig. 6). Together with our previous data we conclude that reaching low FoxP2 levels is a sign that adult-born born MSN in Area X have reached maturity by 6 weeks after their generation in the VZ^34^.

Since our previous work demonstrated that new MSNs participate in singing-associated neural activity in Area X^34^ here we asked if this was linked to FoxP2 levels. After singing FoxP2 mRNA and protein levels are lower in Area X tissue^25–27^ than when birds were silent whereas the expression of the immediate early gene EGR-1 increases linearly the more birds sing undirected song^40,41^. Analyzing expression levels in individual neurons revealed that only FoxP2^low^ MSNs, but not Foxp2^high^ MSNs seemed to be subject to singing induced FoxP2 downregulation^24^. We therefore hypothesized that FoxP2^high^ MSNs were not yet connected into the circuit and therefore not regulated by singing. Our data contradict this hypothesis. We show that the induction of the immediate early gene EGR-1 in MSNs after singing was equally likely in FoxP2^low^ and FoxP2^high^ MSNs, suggesting that both were functionally incorporated into the song circuit.

Previous work showed that the degree of FoxP2 downregulation correlates with the quantity of produced song and depends on auditory feedback, which is relayed to Area X via the cortical song nucleus HVC^27,45^. In our study, the relationship between FoxP2 downregulation and song quantity was present in new MSNs at 42 dpi but not earlier and we therefore suggest that new MSNs start to receive auditory feedback signals between 31 and 42 dpi.

What might cause the age-dependent FoxP2 downregulation in new MSNs? One possibility is that intrinsic mechanisms, depending more on cell age than on extracellular inputs, downregulate FoxP2 during maturation. Another possibility is that EGR-1 gradually decreases FoxP2 levels during every singing event. This latter scenario is consistent with the findings that the *FoxP2* promoter contains EGR-1 binding sites^4,46^ and that EGR-1 expression is crucial for functional integration of new neurons in the adult rodent hippocampus^47^.

We also tested if varying FoxP2 levels affect neuronal morphology in adult male zebra finches and analyzed dendrite complexity and spine density of virally labelled new FoxP2^low^ and FoxP2^high^ MSNs. We now show differences between the morphology of adult generated MSNs that express FoxP2 at high or low levels; high FoxP2 levels were associated with greater dendrite complexity and higher dendritic spine density in comparison to neurons with low FoxP2 levels (Fig. 6). Concerning spine density, our results are consistent with previous studies in juvenile zebra finches, since experimental FoxP2 knockdown decreased overall spine densities of new Area X MSNs^48^. Moreover, our results are in line with findings in mice that link Foxp2 to neuronal outgrowth and spine density in striatal neurons and their progenitors^18,19,35–37^. Additionally, we find similarities between mice and birds on the level of spine types. We show that FoxP2^low^ new MSNs 31 dpi have fewer mushroom spines than FoxP2^high^ new MSNs. In mice, striatal spiny neurons of heterozygous Foxp2 knockdown mice show specifically a decrease of mushroom and branched spines whereas stubby and thin spines are not affected^19^. In birds and mammals, dendritic spines of striatal MSNs receive both glutamatergic dopaminergic input from cortical/pallial regions and the midbrain, respectively. In the case of new MSNs in Area X, we hypothesize that high FoxP2 expression levels during their maturation might increase their capacity for receiving both inputs.

What mechanisms might account for morphological differences between FoxP2^low^ and FoxP2^high^ MSNs? They may originate from differential target gene activation in FoxP2^low^ and FoxP2^high^ MSNs. FoxP2 has hundreds of downstream target genes of which many are part of networks associated with neurite development^35,49–52^. One specific candidate is the myocyte enhancer factor 2C (Mef2c), a negative regulator of synaptogenesis^19^. Foxp2 specifically promotes corticostriatal synaptogenesis via the repression of Mef2c. Whether a similar mechanism shapes the integration of new MSNs into the avian corticostriatal network remains to be elucidated by future studies. In zebra finch Area X, two direct FoxP2 targets are associated with neuronal outgrowth; the very-low-density-lipoprotein receptor (VLDLR) and the contactin-associated protein-like 2 (CNTNAP2). Their expression correlates positively with FoxP2 in juveniles and in singing adults^53,54^. Thus, in new Area X neurons, VLDLR and CNTNAP2 would be expected to be highly activated during singing in FoxP2^high^ MSN but not in FoxP2^low^ MSNs and may thus generate the diverging MSNs morphology we found.

We would like to propose some speculations regarding possible functions of two MSNs subpopulations that differ in FoxP2 expression levels. We found that these populations differed in nucleus size, dendritic complexity and spine density during an early time period of their integration into Area X. We do not know if the observed morphological differences persist long-term because of a lack of markers that could distinguish former FoxP2^high^ MSNs from former FoxP2^low^ MSNs in later maturation phases. If these two subpopulations persist long-term, they might resemble striato-nigral and striato-pallidal MSNs of the direct and indirect pathway in mammals. These MSNs subtypes are morphologically and neurochemically different. Direct pathway MSNs express the dopamine receptor D1 and their dendrites are more complex than indirect pathway MSNs that express the dopamine receptor D2^39,55^. High Foxp2 levels in D1 MSNs and low Foxp2 in D2 MSNs have been proposed to be linked to this anatomical dichotomy^35^. The avian direct and indirect pathway through the basal ganglia however is not characterized by different MSN projections but rather by direct and indirect pallidal-like output neurons that project from Area X to the thalamus^56^. To date, it is not known if MSN subtypes exclusively synapse on either direct or indirect pallidal-like neurons^57^. Contrary to mammalian MSNs, more than half of the Area X MSNs express multiple dopamine receptors^58^ so that they cannot be used as markers for indirect versus direct pathway neurons. Investigating potential avian MSNs subtypes and the developmental role FoxP2 plays in those will be of interest for future studies.

What might be the function of new MSNs in Area X and how is it affected by FoxP2 expression levels? Our previous study showed that once matured, new MSNs have similar characteristics as older, resident MSNs and are active during singing^34^. General MSNs function is feed forward inhibition within a cortico-striatal-thalamo-cortical loop during singing^59^. We hypothesize that new MSNs in adult zebra finches are entrained to produce a correct firing pattern in a plastic phase during their maturation and thus may counteract song drift, as has been suggested before^33,60^. This process might be in influenced by varying FoxP2 expression levels and the resulting morphological differences between putative subpopulations of new MSNs. FoxP2^high^ new neurons with a higher dendrite complexity and more dendritic spines might be more receptive to tuning than FoxP2^low^ new neurons. For further interpretations of our findings it will be crucial to gain additional knowledge on the microcircuitry of Area X and on the role neurons play for its function.

In summary, FoxP2 expression levels vary in adult-born MSN at different maturation times after they have been recruited to Area X. We show that the different FoxP2 expression levels correlate with neuronal morphology and spine density. Varying FoxP2 expression levels during a specific time window might permit different target gene activation important for correct incorporation and function of new MSNs in Area X.

## Methods

### FoxP2 nomenclature

We follow the nomenclature proposed by^61^, *FOXP* refers to the human gene, *Foxp* refers to the mouse gene and *FoxP* refers to all other species. FOXP, Foxp2 and FoxP2 correspond to the protein product.

### Animals

42 adult male zebra finches (*Taeniopygia guttata*, age >120 days) were used in the present study, bred and housed at the Department of Animal Behaviour at Freie Universität Berlin. The colony was kept under a 12:12 h light:dark-cycle with food and water ad libitum. All experiments were reviewed and approved by the veterinary department of the Freie Universität Berlin and by the ethics committee of the Regional Office for Health and Social Affairs Berlin and were performed in accordance with relevant guidelines and regulations. The permit numbers are G0116/13 and G0296/15.

### Experiments

We conduced three experiments. In the first, we analyzed FoxP2 expression levels of new neurons (BrdU+, see below) in Area X at three time points, e.g. at 21 days (5 birds, 166 neurons), 31 days (6 birds, 295 neurons) or 42 days (6 birds, 272 neurons) after BrdU injections (dpi). In the second experiment we analyzed FoxP2 expression levels and the expression of the early growth response protein 1 (EGR-1) at 21 dpi (6 birds, 127 neurons), 31 dpi (6 birds, 108 neurons) and at 42 dpi (6 birds 156 neurons). In the third experiment we analyzed FoxP2 expression levels, dendrite morphology and spine density of new neurons that were labelled via lentiviral infection. In total we analyzed 52 neurons of 4 zebra finches (13 ± 3 neurons/bird, mean ± SD) at 31 dpi and 23 neurons of 3 zebra finches at 42 dpi (7.6 ± 0.5 neurons/bird, mean ± SD).

### BrdU injections

For the analysis of FoxP2 levels and EGR-1 expression in newborn neurons 35 birds received BrdU (50 µg/g) via intramuscular injections in the mornings for 5 consecutive days. Birds were assigned to three groups with different survival times (21, 31, and 42 days after BrdU injection, dpi).

### Song monitoring

For FoxP2 expression level analysis after BrdU treatment or lentiviral injections, 17 birds were isolated in sound attenuated chambers for one night before sacrifice. In the following morning, birds were kept from singing by the experimenter sitting nearby for 1.5 h after lights went on. For EGR-1 expression analysis in new neurons after singing, 18 birds were kept in sound attenuated chambers for three nights and were perfused in the morning of the 4th day 1.5 h after the lights went on. During those 1.5 h birds had to sing at least 150 motifs to be included in the subsequent analysis of EGR-1 expression. Vocalizations were continuously monitored via Sound Analysis Pro^62^.

### Lentiviral Vector injection

To label progenitor cells at the lateral wall of the ventricle, the lentiviral expression vector pFUGW^63^ containing a GFP reporter gene was generated as described in Lois *et al*.^63^ and stereotactically injected into the ventricular zone of 7 birds under isoflurane anesthesia. Titers ranged from 2 × 10^6^ and 3 × 10^7^ viral particles/µl. Birds were fixed in a stereotaxic head holder, with the beak in a 45° angle from the vertical axis. In each hemisphere, approximately 200 µl of virus containing solution were injected into four sites. Following coordinates relative to the bifurcation of the midsagittal sinus were used: anterior-posterior 3.8–4.1, medial-latera −1.3/+1.3, dorsal-ventral −5.0, injection angle: 10° lateral.

### Immunohistochemistry

For immunohistochemical staining birds were overdosed with isoflurane and immediately perfused transcardially with phosphate-buffered saline (PBS) followed by 4% paraformaldehyde (PFA) in PBS. Brains were dissected, post-fixed in 4% PFA for one night and washed for another night in PBS. Brains were cut sagitally or frontally into 50 µm sections using a vibrating microtome (VT1000S, Leica). BrdU antigen retrieval required incubation in 2N HCl for 30 min at 37 °C and neutralization with borate buffer. GFP signal was enhanced via antibody staining. All immunostainings were performed according to standard protocols. The following primary antibodies were used: anti FoxP2 (goat, Abcam ab1307, dilution:1:1000), anti EGR-1 (rabbit, Santa Cruz sc-189, dilution:1:600), anti BrdU (rat, Bio-Rad MCA2060, dilution: 1:200), anti GFP (rabbit, Abcam ab290, dilution:1:1000). Fluorescent secondary antibodies were the following: anti-rat-Alexa-Fluor-488 (Life Technologies, A21208, dilution: 1:200), anti-rabbit-Alexa-Fluor-568 (Life Technologies, A10042, dilution: 1:200), anti-goat-Alaxa-Fluor-647 (Life Technologies, A21447, dilution: 1:200). To visualize nuclei, all sections were counterstained with 4′, 6-Diamidin-2-phenylindol (DAPI, Serva).

### Confocal imaging and image processing and quantification

Z-Stacks of BrdU+ or GFP+ cells in Area X were obtained with a SP8 confocal microscope (Leica). For FoxP2 scanning, all microscope settings were kept constant. Scans of BrdU+ nuclei were performed using a 63x lens (digital zoom 2.0), an image size of 1024 × 1024 pixels and a z-stack size of 1 µm. Whole neurons (GFP+) were imaged using a 63x lens, an image size of 2042 × 2042 pixel and a z-stack size of 1 µm. Acquired images were processed using the Fiji software package^64^. Only neurons with spiny long dendrites were included in the analysis. The Rolling Ball Background Subtraction plugin was used to subtract background. We measured the mean pixel intensity of nuclear FoxP2 expression, by positioning a circle of 4 µm in diameter (12.56 µm^2^) in the center of the BrdU+ nucleus. In total we analyzed the intensity of the FoxP2 expression dependent fluorescence of 166 BrdU+ cells at 21 dpi (n = 5), 295 BrdU+ cells at 31 dpi (n = 6) and 272 BrdU+ cells at 42 dpi (n = 6). FoxP2^high^ were defined as cells that fell into the top 30% of measured mean pixel intensities in one animal (i.e. if the highest mean pixel intensity in one animal was 240 we counted all BrdU+ cells that had a FoxP2 mean pixel intensity between 168 and 240 as FoxP2^high^ neurons). We decided on the 30% value because it covered the FoxP2^high^ expressing cells in the bimodal distribution of all FoxP2 intensities. We defined the neurons that fell into the bottom 30% of measured mean pixel intensities as FoxP2^low^. As for the FoxP2^high^ cutoff, the bottom 30% contained the low-intensity peak of the bimodal density distribution. Because we particularly wanted to address the effect of high and low FoxP2 expression levels on neuronal properties, neurons with intermediate FoxP2 expression levels were not considered for further analysis. The Simple Neurite Tracer plugin (Fiji) was used to trace individual neurons and we analyzed their total branch length and number of primary dendrites. The traces were then used by the Sholl analysis plugin in Fiji^65^. We measured intersections of dendrites with concentric circles that were placed every 10 µm starting from the center of the soma. The maximal number of intersections per neuron was extracted from the Sholl analysis dataset. For dendritic spine analysis images were deconvolved using the Tikhonov-Miller algorithm in the DeconvolutionLab plugin in Fiji^66^. Prior to deconvolution an individual point spread function was generated for each image using the Born and Wolf 3D optical model in the PSF Generator plugin in Fiji^67^. Semiautomated dendritic spine counts were performed using the software NeuronStudio^68^ that uses a spine classification algorithm. For spine classification, the default settings were used to classify spines as mushroom, stubby or thin spines: a head-to-neck ratio threshold of 1.1 µm, a height-to-width ratio threshold of 2.5 µm and a minimum mushroom head size of 0.35 µm. A spine is considered mushroom if the head-to-neck ratio is above the threshold and its head is larger than 0.35 µm. A spine is considered stubby if its head-to-neck ratio and its heights-to-width ratio are below threshold. In all other cases spines were classified as thin. On average, we analyzed spines densities on secondary dendrites along the length of 118 µm ± 1.92 (mean, SEM) per neuron. In total, we analyzed spines of 52 individual neurons of 4 animals in experimental group 31 dpi, and 23 neurons of 3 animals at 42 dpi. Additionally, we measured the dendrite diameter of 44 new neurons in 4 animals (8–12 neurons per animal) using the line measuring tool in Fiji^64^. We took 5 measures on each of 3 secondary dendrites per neuron (in total 15 measures per neuron). The experimenter was blind to FoxP2 levels of individual neurons during the whole quantification process, because cells were selected for quantification based on their BrdU+ fluorescence or their EGR-1 fluorescence and FoxP2 fluorescence in a different channel was quantified last. The datasets generated and analysed during the current study are available from the corresponding author on request.

### Statistics

The software R was used to analyze data^69^. Significance level was p < 0.05 for all tests. Plots were generated using the ggplot package in R^70^. For the analysis of FoxP2^high^ neurons we used a Kruskal-Wallis test followed by a Dunn’s test for pairwise comparison. For the analysis of FoxP2^low^ neurons we used ANOVA followed by a Bonferroni’s multiple comparison test. The relationship of (a) FoxP2 levels and nuclear diameter as well as (b) FoxP2+ neurons and singing were determined using a linear regression analysis. Dendritic spine data (all spines) and Sholl data were analyzed using the paired Student’s t-test. Data from the spine type analysis, mushroom head size, total dendrite length, number of primary dendrites and dendrite thickness were analyzed using the Mann Whitney test. Number of maximal intersections was analyzed using the Student’s t-test. Choice of test was based on previous analysis for normality using the Shapiro-Wilk-test and variance analysis using the F-test or Levene’s test.

## References

[1] Lai CS, Fisher SE, Hurst JA, Vargha-Khadem F, Monaco AP (2001). A forkhead-domain gene is mutated in a severe speech and language disorder. Nature.

[2] Deriziotis P, Fisher SE (2017). Speech and Language: Translating the Genome. Trends Genet.

[3] Morgan, A., Fisher, S. E., Scheffer, I. & Hildebrand, M. In *GeneReviews* (eds Adam, M. P. *et al*.) (2016).

[4] Wohlgemuth S, Adam I, Scharff C (2014). FoxP2 in songbirds. Curr Opin Neurobiol.

[5] Teramitsu I, Kudo LC, London SE, Geschwind DH, White SA (2004). Parallel FoxP1 and FoxP2 expression in songbird and human brain predicts functional interaction. J Neurosci.

[6] Lai CS, Gerrelli D, Monaco AP, Fisher SE, Copp AJ (2003). FOXP2 expression during brain development coincides with adult sites of pathology in a severe speech and language disorder. Brain.

[7] Haesler S (2004). FoxP2 expression in avian vocal learners and non-learners. J Neurosci.

[8] Heston JB, White SA (2015). Behavior-Linked FoxP2 Regulation Enables Zebra Finch Vocal Learning. J Neurosci.

[9] Haesler S (2007). Incomplete and inaccurate vocal imitation after knockdown of FoxP2 in songbird basal ganglia nucleus Area X. PLoS Biol.

[10] Norton P, Barschke P, Scharff C, Mendoza E (2019). Differential Song Deficits after Lentivirus-Mediated Knockdown of FoxP1, FoxP2, or FoxP4 in Area X of Juvenile Zebra Finches. J Neurosci.

[11] Vargha-Khadem F (1998). Neural basis of an inherited speech and language disorder. Proc Natl Acad Sci USA.

[12] Day Nancy F., Hobbs Taylor G., Heston Jonathan B., White Stephanie A. (2019). Beyond Critical Period Learning: Striatal FoxP2 Affects the Active Maintenance of Learned Vocalizations in Adulthood. eneuro.

[13] Murugan M, Harward S, Scharff C, Mooney R (2013). Diminished FoxP2 levels affect dopaminergic modulation of corticostriatal signaling important to song variability. Neuron.

[14] Tsui D, Vessey JP, Tomita H, Kaplan DR, Miller FD (2013). FoxP2 regulates neurogenesis during embryonic cortical development. J Neurosci.

[15] Garcia-Calero E, Botella-Lopez A, Bahamonde O, Perez-Balaguer A, Martinez S (2016). FoxP2 protein levels regulate cell morphology changes and migration patterns in the vertebrate developing telencephalon. Brain Struct Funct.

[16] Clovis YM, Enard W, Marinaro F, Huttner WB, De Pietri Tonelli D (2012). Convergent repression of Foxp2 3′UTR by miR-9 and miR-132 in embryonic mouse neocortex: implications for radial migration of neurons. Development.

[17] Kast, R. J., Lanjewar, A. L., Smith, C. D. & Levitt, P. FOXP2 exhibits projection neuron class specific expression, but is not required for multiple aspects of cortical histogenesis. *Elife* **8**, 10.7554/eLife.42012 (2019). 10.7554/eLife.42012 PMC6561705 31099752

[18] Chiu YC (2014). Foxp2 regulates neuronal differentiation and neuronal subtype specification. Dev Neurobiol.

[19] Chen Yi-Chuan, Kuo Hsiao-Ying, Bornschein Ulrich, Takahashi Hiroshi, Chen Shih-Yun, Lu Kuan-Ming, Yang Hao-Yu, Chen Gui-May, Lin Jing-Ruei, Lee Yi-Hsin, Chou Yun-Chia, Cheng Sin-Jhong, Chien Cheng-Ting, Enard Wolfgang, Hevers Wulf, Pääbo Svante, Graybiel Ann M, Liu Fu-Chin (2016). Foxp2 controls synaptic wiring of corticostriatal circuits and vocal communication by opposing Mef2c. Nature Neuroscience.

[20] Groszer M (2008). Impaired synaptic plasticity and motor learning in mice with a point mutation implicated in human speech deficits. Curr Biol.

[21] French CA (2012). An aetiological Foxp2 mutation causes aberrant striatal activity and alters plasticity during skill learning. Mol Psychiatry.

[22] French CA (2019). Differential effects of Foxp2 disruption in distinct motor circuits. Mol Psychiatry.

[23] van Rhijn JR, Fisher SE, Vernes SC, Nadif Kasri N (2018). Foxp2 loss of function increases striatal direct pathway inhibition via increased GABA release. Brain Struct Funct.

[24] Thompson CK (2013). Young and intense: FoxP2 immunoreactivity in Area X varies with age, song stereotypy, and singing in male zebra finches. Front Neural Circuits.

[25] Teramitsu I, White SA (2006). FoxP2 regulation during undirected singing in adult songbirds. J Neurosci.

[26] Miller JE (2008). Birdsong decreases protein levels of FoxP2, a molecule required for human speech. J Neurophysiol.

[27] Teramitsu I, Poopatanapong A, Torrisi S, White SA (2010). Striatal FoxP2 is actively regulated during songbird sensorimotor learning. PLoS One.

[28] Alvarez-Buylla A, Kirn JR, Nottebohm F (1990). Birth of projection neurons in adult avian brain may be related to perceptual or motor learning. Science.

[29] Alvarez-Buylla A, Ling CY, Yu WS (1994). Contribution of neurons born during embryonic, juvenile, and adult life to the brain of adult canaries: regional specificity and delayed birth of neurons in the song-control nuclei. J Comp Neurol.

[30] Lipkind D, Nottebohm F, Rado R, Barnea A (2002). Social change affects the survival of new neurons in the forebrain of adult songbirds. Behav Brain Res.

[31] Rochefort C, He X, Scotto-Lomassese S, Scharff C (2007). Recruitment of FoxP2-expressing neurons to area X varies during song development. Dev Neurobiol.

[32] Barnea A, Pravosudov V (2011). Birds as a model to study adult neurogenesis: bridging evolutionary, comparative and neuroethological approaches. Eur J Neurosci.

[33] Pytte CL (2016). Adult Neurogenesis in the Songbird: Region-Specific Contributions of New Neurons to Behavioral Plasticity and Stability. Brain Behav Evol.

[34] Kosubek-Langer J, Schulze L, Scharff C (2017). Maturation, Behavioral Activation, and Connectivity of Adult-Born Medium Spiny Neurons in a Striatal Song Nucleus. Front Neurosci.

[35] Vernes SC (2011). Foxp2 regulates gene networks implicated in neurite outgrowth in the developing brain. PLoS Genet.

[36] Enard W (2009). A humanized version of Foxp2 affects cortico-basal ganglia circuits in mice. Cell.

[37] Reimers-Kipping S, Hevers W, Paabo S, Enard W (2011). Humanized Foxp2 specifically affects cortico-basal ganglia circuits. Neuroscience.

[38] Stanley Geoffrey, Gokce Ozgun, Malenka Robert C., Südhof Thomas C., Quake Stephen R. (2020). Continuous and Discrete Neuron Types of the Adult Murine Striatum. Neuron.

[39] Gertler TS, Chan CS, Surmeier DJ (2008). Dichotomous anatomical properties of adult striatal medium spiny neurons. J Neurosci.

[40] Jarvis ED, Scharff C, Grossman MR, Ramos JA, Nottebohm F (1998). For whom the bird sings: context-dependent gene expression. Neuron.

[41] Mello CV, Ribeiro S (1998). ZENK protein regulation by song in the brain of songbirds. J Comp Neurol.

[42] Knapska E, Kaczmarek L (2004). A gene for neuronal plasticity in the mammalian brain: Zif268/Egr-1/NGFI-A/Krox-24/TIS8/ZENK?. Prog Neurobiol.

[43] Hessler NA, Doupe AJ (1999). Social context modulates singing-related neural activity in the songbird forebrain. Nat Neurosci.

[44] Zengin-Toktas Y, Woolley SC (2017). Singing modulates parvalbumin interneurons throughout songbird forebrain vocal control circuitry. PLoS One.

[45] Schmidt MF, Konishi M (1998). Gating of auditory responses in the vocal control system of awake songbirds. Nat Neurosci.

[46] Warren WC (2010). The genome of a songbird. Nature.

[47] Veyrac A (2013). Zif268/egr1 gene controls the selection, maturation and functional integration of adult hippocampal newborn neurons by learning. Proc Natl Acad Sci USA.

[48] Schulz SB, Haesler S, Scharff C, Rochefort C (2010). Knockdown of FoxP2 alters spine density in Area X of the zebra finch. Genes Brain Behav.

[49] Spiteri E (2007). Identification of the transcriptional targets of FOXP2, a gene linked to speech and language, in developing human brain. Am J Hum Genet.

[50] Vernes SC (2007). High-throughput analysis of promoter occupancy reveals direct neural targets of FOXP2, a gene mutated in speech and language disorders. Am J Hum Genet.

[51] Hickey SL, Berto S, Konopka G (2019). Chromatin Decondensation by FOXP2 Promotes Human Neuron Maturation and Expression of Neurodevelopmental Disease Genes. Cell Rep.

[52] Konopka G (2009). Human-specific transcriptional regulation of CNS development genes by FOXP2. Nature.

[53] Adam I, Mendoza E, Kobalz U, Wohlgemuth S, Scharff C (2016). FoxP2 directly regulates the reelin receptor VLDLR developmentally and by singing. Mol Cell Neurosci.

[54] Adam I, Mendoza E, Kobalz U, Wohlgemuth S, Scharff C (2017). CNTNAP2 is a direct FoxP2 target *in vitro* and *in vivo* in zebra finches: complex regulation by age and activity. Genes Brain Behav.

[55] Calabresi P, Picconi B, Tozzi A, Ghiglieri V, Di Filippo M (2014). Direct and indirect pathways of basal ganglia: a critical reappraisal. Nat Neurosci.

[56] Farries MA, Ding L, Perkel DJ (2005). Evidence for “direct” and “indirect” pathways through the song system basal ganglia. J Comp Neurol.

[57] Pidoux M, Bollu T, Riccelli T, Goldberg JH (2015). Origins of basal ganglia output signals in singing juvenile birds. J Neurophysiol.

[58] Kubikova L, Wada K, Jarvis ED (2010). Dopamine receptors in a songbird brain. J Comp Neurol.

[59] Perkel DJ, Farries MA, Luo M, Ding L (2002). Electrophysiological analysis of a songbird basal ganglia circuit essential for vocal plasticity. Brain Res Bull.

[60] Wilbrecht L, Kirn JR (2004). Neuron addition and loss in the song system: regulation and function. Ann N Y Acad Sci.

[61] Kaestner KH, Knochel W, Martinez DE (2000). Unified nomenclature for the winged helix/forkhead transcription factors. Genes Dev.

[62] Tchernichovski O, Nottebohm F, Ho CE, Pesaran B, Mitra PP (2000). A procedure for an automated measurement of song similarity. Anim Behav.

[63] Lois C, Hong EJ, Pease S, Brown EJ, Baltimore D (2002). Germline transmission and tissue-specific expression of transgenes delivered by lentiviral vectors. Science.

[64] Schindelin J (2012). Fiji: an open-source platform for biological-image analysis. Nat Methods.

[65] Ferreira TA (2014). Neuronal morphometry directly from bitmap images. Nat Methods.

[66] Sage D (2017). DeconvolutionLab2: An open-source software for deconvolution microscopy. Methods.

[67] Kirshner H, Aguet F, Sage D, Unser M (2013). 3-D PSF fitting for fluorescence microscopy: implementation and localization application. J Microsc.

[68] Rodriguez A, Ehlenberger DB, Dickstein DL, Hof PR, Wearne SL (2008). Automated three-dimensional detection and shape classification of dendritic spines from fluorescence microscopy images. PLoS One.

[69] R: A Language and Environment for Statistical Computing (R Foundation for Statistical Computing, 2013).

[70] Wickham, H. *gglot2: Elegant Graphics for Data Analysis*. (Springer, 2016).

